# Cellular and proteomic differences associated with lithium response in olfactory neuroepithelium cells of bipolar disorder patients

**DOI:** 10.1186/s10020-025-01343-x

**Published:** 2025-09-26

**Authors:** Maria Hidalgo-Figueroa, Alejandra Delgado-Sequera, Anaid Pérez-Ramos, MªCarmen Durán-Ruiz, Cristina Romero-Lopez-Alberca, Jose I. Pérez-Revuelta, Ingrid Marquez-Estefenn, Clara García-Mompó, Jose Ma Villagrán Moreno, Esther Berrocoso

**Affiliations:** 1 Neuropsychopharmacology and Psychobiology Research Group, University of Cadiz, Cádiz, Spain; 2 Biomedical Research and Innovation Institute of Cadiz (INiBICA), Cádiz, Spain; 3 Centre for Biomedical Research in Mental Health (CIBERSAM), Instituto de Salud Carlos III, Madrid, Spain; 4 Department of Psychology, University of Cadiz, Cádiz, Spain; 5 Biomedicine, Biotechnology and Public Health Department, University of Cadiz, Cádiz, Spain; 6 Department of Mental Health, Jerez de la Frontera University Hospital, Cádiz, Spain; 7 Severe Mental Disorder Research Group, Department of Medicine and Surgery, University of Cadiz, Cádiz, Spain; 8 Department of Otolaryngology, Puerta del Mar University Hospital, Cádiz, Spain; 9 Department of Neuroscience, University of Cadiz, Cádiz, Spain

**Keywords:** Bipolar disorder, Cytoskeleton, Lithium, Olfactory neuroepithelium, Proteomics

## Abstract

**Background:**

Lithium is a first-line treatment for bipolar disorder (BD), but only 30% of patients respond satisfactorily to monotherapy, and the biological basis for this variability remains unclear. This study aimed to identify potential biomarkers and therapeutic targets by analyzing olfactory neuroepithelium (ONE) cells from BD lithium non-responders (BDNR), responders (BDR), and control subjects.

**Methods:**

Immunofluorescence and proteomic analyses of ONE cells were conducted. Blood samples were examined to improve accessibility for clinical applications.

**Results:**

Immunofluorescence and proteomic analyses of ONE cells revealed that BDNR cells exhibited impaired adhesion capacity, which was restored by lithium treatment in vitro. However, BDNR cells also showed significant alterations in cell morphology and cytoskeletal organization that were unaffected by lithium. Proteomic analysis identified significant changes in pathways associated with “cell morphology,” with CDN2A highlighted as a key protein. In BDR cells, lithium treatment restored adhesion capacity but failed to reverse migration deficits. Proteomic analysis of BDR ONE cells identified differentially expressed proteins linked to neurotransmitter release, synaptic function, and mitochondrial activity, many of which were significantly modulated by lithium. Additionally, peripheral blood mononuclear cells from BDR patients displayed lower levels of RHOC protein, mirroring reductions seen in ONE BDR cells treated with lithium.

**Conclusions:**

This study underscores cellular and proteomic differences between BDNR and BDR cells, with lithium exerting pronounced effects on BDR cells while having limited impact on BDNR cells. These findings advance our understanding of lithium responsiveness in BD and point to potential biomarkers and therapeutic targets for personalized treatment approaches.

## Introduction

Bipolar disorder (BD) is a highly heritable psychiatric condition with a lifetime prevalence of 1–3% (Grande et al. 2016). It is characterized by recurrent episodes of (hypo)mania and depression, typically emerging in young adulthood, and often follows a chronic and recurrent course (Carvalho et al. 2020). BD is associated with severe social and neurocognitive functional impairments that negatively affect quality of life and increase the risk of suicide (Plans et al. 2019), making it a significant public health concern and a leading contributor to global disability (Vos et al. 2017).

Lithium, considered the gold-standard treatment for BD, was discovered over 70 years ago through clinical observation, yet its precise mechanism of action remains only partially understood. Despite this, lithium’s efficacy in reducing relapse risk—particularly in preventing manic episodes—and its ability to significantly lower the risk of suicide attempts are well-documented (Fountoulakis et al. 2022; Hayes et al. 2016; Miura et al. 2014; McKnight et al. 2019). However, patient response to lithium varies significantly. Studies consistently show that 20–30% of patients experience sustained improvement, with a marked reduction or even complete cessation of affective episodes. Conversely, around 30% of patients show only partial responsiveness, while over 40% derive no clinical benefit (Borkowska and Rybakowski 2001). These distinct response profiles seem to be associated with specific phenotypic traits, and genetic factors (Lin et al. 2021; Nunes et al. 2022). Nevertheless, efforts to create predictive models based solely on clinical data have so far failed to produce tools of clinical utility. Given this considerable variability in treatment outcomes, the ability to predict individual responses to lithium prior to treatment would be invaluable in reducing the duration of untreated illness in BD patients. Unfortunately, despite decades of research, progress in this area remains limited—likely due to the significant limitations of current cellular, tissue, and animal models, which often lack validity and have poor predictive power regarding drug efficacy (Grof et al., 2002).

Recent advances in cellular models using human stem cells, such as induced pluripotent stem cells (iPSCs) and olfactory neuroepithelium (ONE)-derived neural progenitor cells (NPCs), have opened new avenues for studying BD. These models allow researchers to derive specific brain cell types and study the disorder in a more physiological context. Studies using ONE cells from BD patients have identified several abnormalities, including altered intracellular calcium signaling (Hahn et al. 2005), increased cell death (McCurdy et al. 2006), cytoskeletal defects, reduced adhesion, and disrupted migration patterns (Delgado-Sequera et al. 2024b; Solís-Chagoyán et al. 2013). Additionally, gene and protein analyses in these cells revealed differential expression patterns related to inositol metabolism, apoptosis, cell signaling, and adhesion compared to controls (Delgado-Sequera et al. 2024b; McCurdy et al. 2006). Research using iPSC-derived NPCs and neurons has also uncovered abnormalities in neural patterning, postmitotic calcium signaling, and neuronal excitability in BD patients (Hoffmann et al. 2018). Notably, increased neuronal excitability in iPSC-derived hippocampal neurons from BD patients has been shown to be selectively reduced by lithium in neurons from patients who are clinical lithium responders (Mertens et al. 2015; Stern et al. 2020). These findings suggest that the neurobiological mechanisms underlying BD can be recapitulated in iPSC and ONE cell models, offering promising avenues for identifying biomarkers that could predict treatment response, thus accelerating treatment optimization and advancing precision medicine in psychiatry.

Despite the well-established variability in lithium response, a comprehensive understanding of the distinct cellular and proteomic profiles of BDR and BDNR remains elusive, particularly in readily accessible cell models like the ONE. While iPSC-derived neurons serve as a valuable platform for modeling brain-specific cell types, ONE cells offer a uniquely accessible, neural derived alternative. These cells are relatively easy to obtain, exhibit robust self-renewal in vitro, and—crucially—do not require genetic reprogramming. Notably, they have demonstrated the capacity to recapitulate cellular abnormalities associated with BD, offering several significant advantages (Delgado-Sequera et al. 2024b; Solís-Chagoyán et al. 2013). Thus, ONE cells offer a relevant and practical system for investigating potential peripheral biomarkers of lithium response. This follow-up study builds on the work of Delgado-Sequera et al. (Delgado-Sequera et al. 2024b). Using ONE cell cultures derived from patients with BD type I, characterized by alternating manic and depressive episodes, we seek to expand on these findings. Specifically, this exploratory study aims to investigate cellular and proteomic differences in ONE cells from BDR and BDNR patients, identifying potential biomarkers of lithium response and novel pharmacological targets that could lead to more precise, personalized treatment strategies for BD, ultimately improving patient outcomes and reducing the duration of untreated illness. This involves exploring correlations between in vivo clinical response and in vitro profiles. Based on previous findings of cellular dysregulation in BD and the variable clinical response to lithium, we hypothesize that lithium treatment in vitro will differentially modulate cellular and proteomic profiles in ONE cells derived from BDR and BDNR patients compared to controls. By categorizing patient samples into lithium responders and non-responders, we will gain deeper insights into the complex biological mechanisms that govern lithium responsiveness.

## Materials and methods

### Subjects

A total of 11 patients with BD and 13 control subjects were initially recruited for the study. Recruitment for the BD group was primarily carried out through the Community Unit of Jerez in Cádiz, Spain, with support from patient associations, while the control group was drawn from the general population.

All patients received outpatient care and met the following inclusion criteria: aged 18–70 years at the time of recruitment, diagnosed with BD type I according to DSM-5 criteria (APA, 2013), fluent in Spanish, capable of providing written informed consent, and with a history of at least one year of lithium treatment. Exclusion criteria for both BD patients and controls included: a DSM-5 diagnosis of intellectual disability, a history of head trauma with loss of consciousness, the presence of an organic disease affecting mental health, or conditions impairing the olfactory mucosa (e.g., nasal polyps, sinusitis, rhinitis). Other exclusion criteria included respiratory or digestive symptoms, or SARS-CoV-2 infection at the time of sample collection. Additionally, control participants were excluded if they had first-degree relatives with severe mental disorders. All participants have given written consent and the study was approved by the Andalusian Ethics Committee (CCEIBA). The authors declare that all procedures contributing to this work comply with the ethical standards of relevant guidelines and regulations.

### Sociodemographic and clinical assessment

The diagnosis of BD type I was confirmed using the Structured Clinical Interview for DSM-5 Disorders (First et al. 2016). Sociodemographic, clinical, and pharmacological data were collected for all participants.

Manic and depressive symptoms were assessed using the Spanish validated version of the Young Mania Rating Scale (YMRS) (Colom et al. 2002; Young et al. 1978) and the 17-item Hamilton Depression Rating Scale (HDRS) (Hamilton, 1960; Ramos-Brieva and Cordero Villafáfila 1986), respectively. The response to lithium treatment was evaluated using the Retrospective Criteria of Long-Term Treatment Response in Research Subjects with Bipolar Disorder scale (ALDA) (Grof et al., 2002; Manchia et al. 2013; Schulze et al. 2010). This scale consists of 11 items divided into two main criteria: Criterion A, which measures the change in frequency and severity of affective symptoms, and Criterion B, which assesses the degree of improvement attributed to the treatment. Based on the available literature (Manchia et al. 2013; Marie-Claire et al. 2023), patients with a total score of 7 or higher were classified as good responders (BR), while those with a total score of 3 or lower were categorized as non-responders (BNR).

### Nasal brushing and cell culture

**Fig. 1:**
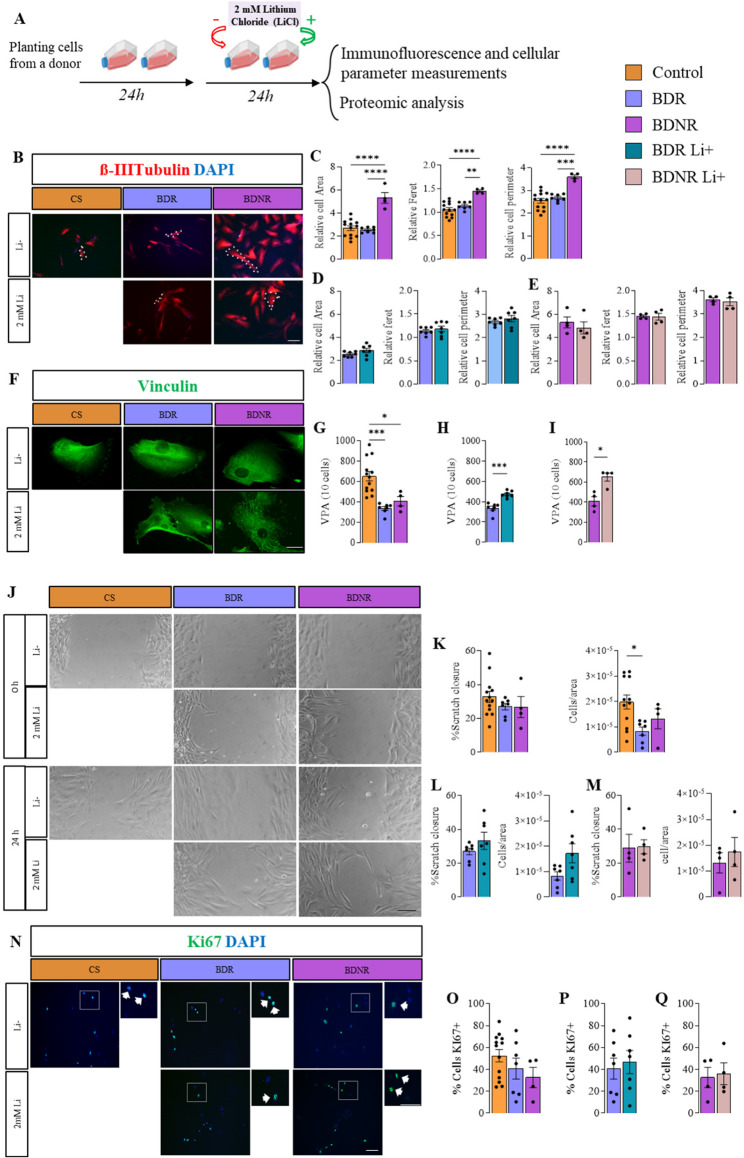
Quantification of cellular parameters. **A** Cell Culture Protocol: Cells were cultured for 48 h, with the last 24 h in the absence or presence of lithium, followed by analysis via immunofluorescence or proteomic assays. **B** Immunocytochemistry: Representative images of β-III Tubulin (red) and DAPI staining (blue). **C** Cell Morphology Quantification: Measurements of cell area, Feret diameter, and perimeter in cells derived from control, BDR, and BDNR groups. **D**-**E** Effect of Lithium on Morphology: Quantification of cell area, Feret diameter, and perimeter in BD patient-derived cells with or without in vitro lithium treatment. **F** Vinculin Immunocytochemistry: Representative images of vinculin staining (green). **G** Vinculin Adhesion Points (VPA): Quantification of VPAs in cells from control, BDR, and BDNR groups. **H**-**I** Lithium Effect on VPAs: Quantification of VPAs in BD patient-derived cells with or without lithium treatment. **J** Wound Healing Assay: Microscope images showing wound closure at 0 and 24 h post-scratch. **K** Cell Migration Analysis: Quantification of scratch closure percentage and number of migrating cells in control, BDR, and BDNR groups after 24 h. **L**-**M** Lithium and Cell Migration: Quantification of scratch closure percentage and migrating cells in BD patient-derived cells treated with or without lithium. **N** Ki67 Immunocytochemistry: Representative images showing Ki67 (green) and DAPI staining (blue). The dashed-line squares indicate the areas shown at higher magnification, where the arrows highlight examples of Ki67 + cells. **O** Proliferation Analysis: Quantification of Ki67 + cells in control, BDR, and BDNR groups. **P**-**Q** Lithium Effect on Proliferation: Quantification of Ki67 + cells in BD patient-derived cells with or without lithium treatment. BDR: bipolar disorder patients responder to lithium; BDNR: bipolar disorder patients non-responder to lithium; Li+: treated with lithium in vitro; Li-: non-treated with lithium in vitro. Statistical Analysis: One-way ANOVA followed by Bonferroni post-hoc test for comparisons among three groups; t-test for comparisons between two groups. *p < 0.05; **p < 0.01; ***p < 0.001; ****<0.0001. Scale bar = 50 μm. Error bars indicate SEM

Cells were exfoliated from the nasal cavity as described previously (Delgado-Sequera et al. 2021, 2024b). Using specialized brushes, samples were collected from the lower and middle turbinate, as well as the nasal cavity roof, through a circular motion. Cells were grown at 37 °C and with 5% CO_2_ in Dulbecco’s Modified Eagle Medium/Ham F-12 (DMEM/F12) containing 10% fetal bovine serum (FBS), 2% GlutaMAX 100X and 0,2% primocin (Thermo Scientific, Spain). When confluent, the cells were detached with 0.25% trypsin-EDTA (GibcoBRL, USA), and about 200,000 cells were re-plated in 75 cm^2^ flasks and cultured in supplemented medium. Independent cell cultures were performed for each subject. All experiments were carried out on cells cultured to passage 6. The following experiments were conducted with and without lithium. A 2 mM concentration of lithium chloride (LiCl, Sigma, Spain) was applied for 24 h, following established protocols from previous studies, to ensure an appropriate balance between efficacy and toxicity (Barbisan et al. 2018; Chen et al. 2014; De Paula et al. 2016; Kim et al. 2021; Wu et al., 2023) (Fig. 1A).

### Collection of human blood samples

Venous blood samples (10 mL) were collected in the morning after overnight fasting. Blood tubes were centrifuged at 1000 g for 10 min at 4 °C, and the resulting plasma samples were stored at −80 °C. PBMCs were then isolated by density gradient centrifugation (30 min at 900×g; Ficoll^®^ Paque Plus, GE HealtCSare Life Sciences, Pittsburgh, PA, USA). The pellet was stored at −80 °C until use.

### Immunocytochemistry

Cells were plated on 13-mm round glass coverslips and incubated at 37 °C in 5% CO_2_ prior to fixation for immunofluorescence, performing all experiments in triplicate: 1 × 10^4^ cells/coverslip. To explore the effect of lithium treatment in vitro, BDR and BDNR ONE cells were treated with 2 mM LiCl (Sigma, Spain) (Fig. 1A). The cells were fixed with 4% paraformaldehyde (PFA: Sigma, Spain) in PBS for 20 min and after washing in PBS, the cells were incubated for 1 h at room temperature (RT) in blocking solution: 10% Bovine Serum Albumin (BSA: Sigma, Spain); 0.25% Triton-X-100; and 1% FBS in PBS. The cells were then incubated overnight at 4 °C with the primary antibodies. Neuronal microtubules were stained with mouse anti-β-III-Tubulin (1:1000 dilution: ThermoFisher (MA1-118), USA). When anti-Ki67 was used, the cells were permeabilised with 0.2% Triton-X 100 in PBS for 25 min and then incubated for 20 min in blocking solution containing 4% FBS. The cells were incubated overnight at 4 °C with anti-Ki67 (1:100 dilution: ThermoFisher, USA) in a buffer containing 0.03% Triton-X 100 and 3% BSA in PBS. The cells were incubated with the secondary antibodies for 1 h at RT: AlexaFluor488 donkey anti-Rabbit (1:1000 dilution: Invitrogen (A21206, USA)) and AlexaFluor568 donkey anti-Mouse (1:1000 dilution: Invitrogen (A10037, USA)). The nuclei were stained with 4′, 6′-diamidino-2-phenylindole dihydrochloride (DAPI, 1:5000 dilution: Panreac Applichem (A4099), Spain), and all the coverslips were then mounted with Fluoro-Gel medium (Electron Microscopy Sciences, USA). Images were acquired on an MMI CellCut plus (Olympus, Japan).

### Cell size and shape measurement

Neuronal microtubules were identified by β-III-tubulin immunostaining, as described above and in triplicate cultures for each sample. A total of 100 cells were assessed per sample from 15 random microscopy fields. The area and perimeter occupied by microtubules were defined and measured with the ImageJ software (USA).

### Focal adhesion Immunolabelling

To analyse focal adhesion, 2 × 10^3^ cells were plated on a coverslip as indicated in the datasheet for the “actin cytoskeleton and focal adhesion staining kit” (Merck (FAK100), Spain). To explore the effect of lithium treatment in vitro on focal adhesion, BDR and BDNR ONE cells were treated with 2 mM LiCl (Sigma, Spain). Cells were permeabilized with 0.5% Triton-X 100 in PBS for 30 min and then incubated in blocking solution for 1 h at RT, containing 1% BSA. The primary anti-vinculin antibody (1:500 dilution: Merck (90227), Spain) was applied overnight (4 °C) and after washing (5 min) in three times, the cells were incubated for 1 h at RT with TRITC-conjugated Phalloidin (1:1000, Merck (90228), Spain) and an AlexaFluor488 Donkey anti-Mouse secondary antibody (1:1000 dilution; ThermoFisher (A21202), USA). Images were acquired with an MMI CellCut plus (Olympus, Japan). To quantify focal adhesions immunolabelled with anti-vinculin, images were analysed with ImageJ software (USA), and the total number of vinculin adhesion points were quantified per cell. The cell cultures were performed in triplicate and a total of 10 cells per sample were analysed.

### Wound healing assay

The assay was performed as indicated in the datasheet for the “Wound Healing Assay” (Ibidi, Germany). A 70 µl suspension of a 3 × 10^5^ cells was added into each well of the 2-Well Culture-Insert and after 24 h, when the cell monolayer was confluent, the 2-Well Culture-Insert was removed and fresh medium was added. At that time (0 h), LiCl (2mM, Sigma, Spain) was added to the medium and a scratch was made in the culture, which was free of cells, and images were taken at 0 and 24 h later, to quantify the number of cells migrating into the wound and the percentage of closure. Each sample was studied in triplicate.

### Sample Preparation for proteomic analysis

ONE cells were cultured under proliferative conditions for 48 h, where in the last 24 h were in vitro treated with 2 mM LiCl (Sigma, Spain) and then lysed (1 × 10^6^ cells per condition) in lysis buffer for protein extraction: 1% NP40, 50 mM HEPES [pH 7], 150 mM NaCl, 1 mM EDTA, supplemented with protease inhibitors. Lysis of PMBCs obtained from patients (approximately 1 × 10^7^ cells) was also performed. Briefly, proteins (100 µg/sample) were precipitated in acetone overnight at − 20 °C, and digested with Trypsin and LysC enzymes (enzyme/substrate ratio 1:50), prior further analysis by mass spectrometry (MS) with a label free quantitative (LFQ) approach, as described (Delgado-Sequera et al. 2024b). Thus, peptide samples (approximately 500 ng/sample) were loaded onto a nano-ACQUITY UPLC System (Waters, USA) connected on-line to an LTQ Orbitrap XL mass spectrometer (Thermo Electron, USA). An aliquot of each sample was loaded onto a Symmetry 300 C18 UPLC Trap column (180 μm × 20 mm, 5 μm: Waters), and the pre-column was connected to a BEH130 C18 column (75 μm × 200 mm, 1.7 μm: Waters, USA) and equilibrated in 3% acetonitrile and 0.1% formic acid. Peptides were analyzed with a 120 min Linear gradient of 3–50% acetonitrile, at 300 nl/min, in a data-dependent acquisition mode. Full MS scan survey spectra (m/z 400–2000) were acquired in the orbitrap with mass resolution of 30,000 at m/z 400.The six most intense ions above 1000 counts were sequentially subjected to collision-induced dissociation (CID) in the linear ion trap. A Progenesis LC-MS (Waters, USA) software was used for data processing and LFQ analysis, using one run as a reference, to which the precursor masses in all the other samples were aligned. Only features comprising charges of 2 + and 3 + were selected, and the raw abundances of each feature were normalized automatically and converted to logarithms against the reference run. A peak list containing the information of all the features was generated and exported to the Mascot search engine (Matrix Science Ltd., UK). Protein-related statistics were obtained with Perseus (v1.6.15.0) software (Tyanova et al. 2016). Briefly, data were log2 transformed, andproteins were filtered to include only those identified in at least 70% of samples.Subsequently, data were normalized and imputed with the normal distribution approach. A two-sample Student’s t-test was performed, and proteins were considered differentially expressed between the groups when *p*-value < 0.01. Together with the fold change, the data were finally uploaded into the Ingenuity^®^ Pathway Analysis (IPA) software (Ingenuity Systems, USA) to investigate their molecular and biological functions.

### Statistical analysis

Statistical analyses were conducted using IBM SPSS Statistics (IBM SPSS Statistics for Windows, Version 29.0. Armonk, NY: IBM Corp) and GraphPad Prism software (GraphPad Software, USA). The normality of data distribution was examined using the Shapiro-Wilk or Kolmogorov-Smirnov tests. Comparisons between two groups were carried out with Student’s t-test when the data followed a normal distribution, or the Mann-Whitney U test for non-normal distributions. For comparisons involving three or more groups, ANOVA was applied for normally distributed data, while the Kruskal-Wallis test was used for non-normal distributions. These were followed by Tukey’s or Dunn’s post hoc tests, respectively, for multiple comparisons. Categorical data were analyzed using Fisher’s exact test.

The area under the Receiver Operating Characteristic (ROC) curve (AUROC) was employed to identify potential biomarkers with discriminative capacity among the expressed proteins. AUROC values reflect the biomarker’s overall discriminative power, where a value of 1 indicates perfect classification ability, and 0.5 represents random classification. To evaluate potential associations between cellular parameters and molecules, correlation coefficients were calculated: Pearson’s for data with normal distribution and Spearman’s for data without normal distribution. A p-value of less than 0.05 was considered statistically significant, unless stated otherwise.

## Results

### Demographic and clinical data of participants

Table 1 summarizes the sociodemographic and clinical characteristics of the control group (*n* = 13) and BD patients (*n* = 11) at the time of biological sample collection. The BD group was further subdivided according to their ALDA score, resulting in 7 BDR patients and 4 BDNR patients.

**Table 1:** Sociodemographic and clinical characteristics

	BDR (*N* = 7)	BDNR (*N* = 4)	C (*N* = 13)	Statistic	*p*
Age, mean (SD)	50 (10)	50 (13)	48 (12)	H	0.889
Sex (N, %)					
Male	4 (57.1)	4 (100)	7 (53.8)		0.381
Female	3 (42.9)	0	6 (46.2)	F	
Education (years), mean (SD)	15 (4)	14 (3)	19 (4)	H	**0.035**
SES, N (%)				F	
Low	5 (71.4)	2 (50)	1 (7.7)		**0.027**
Medium	2 (28.6)	2 (50)	8 (61.5)		
High	0	0	4 (30.8)		
Marital Status, N (%)				F	0.155
Single	3 (42.9)	2 (50)	2 (15.4)		
Married	3 (42.9)	2 (50)	11 (84.6)		
Divorced	1 (14.3)	0	0		
Current Tobacco, N (%)				F	0.638
No	3 (42.9)	1 (25)	7 (58.3)		
Yes	4 (57.1)	3 (75)	5 (41.7)		
Current Cannabis, N (%)				F	1.00
No	7 (100)	4 (100)	10 (83.3)		
Yes	0	0	2 (16.6)		
YMRS, mean (SD)	3.8 (3.2)	6.7 (4.9)	-	U	0.438
HDRS, mean (SD)	4.7 (5.2)	11.2 (7.8)	-	U	0.107
Pharmacological treatment, mean (SD)					
Lithium (mg)	1014 (261)	1050 (100)	-	U	0.482
Valproate (mg)	-	2333.3 (1040.8)	-	U	
Antidepressant (mg)	20 (14.14)	-	-	U	
Antipsychotic (mg)	17.5 (17.6)	187.8 (164.6)	-	U	0.088
Benzodiazepine (mg)	0.14 (0.41)	4.7 (6.06)	-	U	.**028**

Bold numbers indicate statistically significant differences

Abbreviations: *BNR* Lithium non responders,* BR* Lithium responders, *CGI* Clinical Global Impression, *C* Controls, *F* Fisher’s exact test, *H* Kruskal-Wallis test, *HDRS* Hamilton Depression Rating Scale, *Mg * milligrams, *N* Sample Size, *SD* Standard Deviation, *SES* Socioeconomic status, *U* Mann–Whitney–Wilcoxon test, *YMRS* Young Mania Rating Scale

No significant differences were found between the groups regarding age, sex, marital status, tobacco use, or cannabis use. However, significant differences were identified in years of education; specifically, the BDNR group had fewer average years of education compared to the control groups. Socioeconomic status also differed significantly across groups, with Fisher’s exact test showing a higher proportion of individuals in low socioeconomic category within the BDNR group, contrasting with a greater representation in medium and high socioeconomic categories among controls. Manic (YMRS) and depressive symptom (HDRS) severity did not significantly differ between the groups. Regarding pharmacological treatment, no significant differences were observed between the BDR and BDNR groups, except for benzodiazepine doses, which were higher in BDNR patients. The average daily dose of lithium was 1014 mg for BDR patients and 1050 mg for BDNR patients. Only the BDR group was prescribed antidepressants, while none received a combination of lithium and valproate. Conversely, this combination was observed in the BDNR group. Controls were not taking psychotropic medication and showed non-pathological scores on the HDRS and YMRS scales.

### Evaluation of cellular characteristics of ONE cells derived from BDR and BDNR

#### Patients

Patient-derived cells were examined under two conditions: with and without lithium in the culture medium. Lithium treatment was applied during the final 24 h of the culture period (Fig. 1A). We first assessed the cell size of ONE cells by measuring the area occupied by microtubules, visualized through β-III Tubulin staining (Fig. 1B). ONE cells derived from BDNR patients exhibited significantly larger cell sizes compared to those from controls and BDR patients (Fig. 1C). Additionally, BDNR-derived cells showed a significant increase in Feret’s diameter and perimeter, suggesting distinct morphological differences, likely indicative of a larger and more elongated cell shape in BDNR patients.These persistent morphological alterations in BDNR-derived cells may represent specific cellular vulnerabilities or traits associated with a lack of clinical lithium response, potentially offering novel phenotypes for stratification or targets for future therapeutic interventions. In contrast, in vitro lithium treatment (2 mM LiCl) did not affect any of these parameters in either the BDR or BDNR groups (Fig. 1D-E).

Next, we quantified focal adhesions in monolayer cultures of cells immunolabeled with anti-vinculin (Fig. 1F-I). The analysis revealed a significant reduction in vinculin points of adhesion (VPA) in both BDR and BDNR cells compared to controls (Fig. 1G). Interestingly, in vitro lithium treatment significantly increased VPA in both BDR (Fig. 1H) and BDNR cells (Fig. 1I). This restoration of focal adhesion capacity by lithium is notable, especially when contrasted with its failure to reverse the distinct morphological changes observed in BDNR cells. This differential in vitro effect of lithium is significant, underscoring that while lithium can modulate certain cellular deficits associated with BD, its mechanism of action does not encompass all observed cellular abnormalities, particularly those characteristic of non-responders.

To investigate whether the reduction in adhesion was associated with changes in cell migration, a scratch wound assay was performed (Fig. 1J-M). No significant differences were observed in the percentage of scratch closure after 24 h between groups (Fig. 1K). However, quantification of the number of cells migrating into the scratch area showed a reduction in migrating cells in the BDR group compared to controls (Fig. 1K). This reduction was not observed in BDNR cells. Lithium treatment did not produce significant changes in migration parameters in either the BDR or BDNR groups compared to untreated conditions (Fig. 1L-M).

Cell proliferation was assessed via immunostaining for Ki67 (Fig. 1N-Q). Analysis revealed no significant differences in proliferation rates between cells from BD patients and controls (Fig. 1O). Likewise, lithium treatment did not affect the proliferation of cells derived from either the BDR or BDNR groups (Fig. 1P-Q).

#### Analysis of differentially expressed proteins in BDR and BDNR ONE cells

**Fig. 2:**
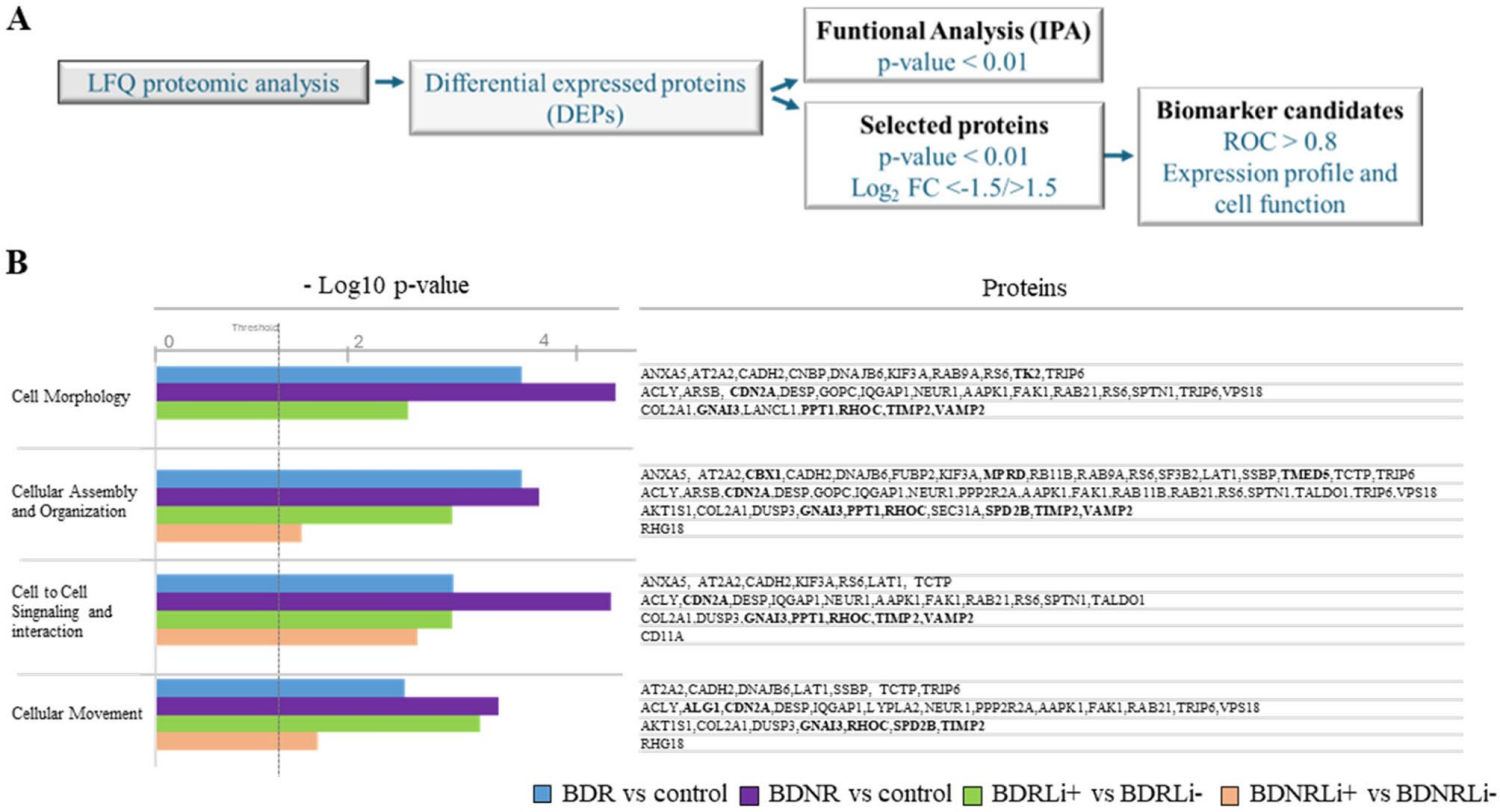
Proteomic analysis. **A** Proteomic analysis workflow for performing functional analysis and identifying biomarker candidates. **B** Altered biofunctions associated with proteomic changes between BDR and control, BDNR and control, BDR Li + and BDR Li-, BDNR Li + and BDNR Li- groups. BDR: bipolar disorder patients responder to lithium; BDNR: bipolar disorder patients non-responder to lithium; Li+: treated with lithium in vitro; Li-: non-treated with lithium in vitro. LFQ: Label free quantitative. Bold proteins: DEPs further analyzed as biomarker candidates (see Fig. 2)

The LFQ proteomic analysis was performed to identify differentially expressed proteins (DEPs) between groups. To this end, ONE cells derived from both BDR and BDNR were compared to cells from controls and incubated in the presence or absence of lithium. Full details on identification and quantification data, including log2 fold changes (FC) and p-values, are provided in Supplementary Table S1. The DEPs were utilized for both functional analysis and the identification of potential biomarker candidates (Fig. 2A).

First, to identify molecular mechanisms altered in BDR and BDNR cells that might explain the cellular alterations described above (Fig. 1), functional analyses were conducted by using IPA software. Full information derived from functional analysis can be found in Supplementary Table S2-S5. The comparisons conducted to identify altered functions included BDR vs. control; BDNR vs. control; BDRLi + vs. BDRLi-; and BDNRLi + vs. BDNRLi-. This analysis revealed several highly significant biological processes and pathways, consistently appearing across multiple group comparisons. Notably, pathways related to cancer were prominently identified, alongside those associated with Cell Death and Survival, Cellular Development, Cellular Growth and Proliferation, and Cellular Movement. Furthermore, pathways implicated in Hereditary Disorder, Neurological Disease, Organismal Injury and Abnormalities, and Infectious Diseases also emerged as highly significant, collectively pointing to fundamental cellular dysregulations pertinent to BD and its response to lithium (Supplementary Table S2-S5). Among the significantly altered functions across all comparison groups we focused on “cell morphology”, “cellular assembly and organization”, “cell-to-cell signalling and interaction”, and “cellular movement” (Fig. 2B). Notably, BDNR vs. control cells exhibited the highest significant variation in the number of proteins associated with each of these functions (-log10 p-value, Fig. 2). While lithium treatment impacted these functions in both BDR and BDNR cells, leading to significant differential expression of numerous proteins, the extent of this variation was notably reduced in BDNR cells post-treatment. This is particularly evident in “cell morphology”, where lithium treatment in BDNR cells resulted in no significant changes compared to BDNR un-treated ones (Fig. 2).

**Fig. 3:**
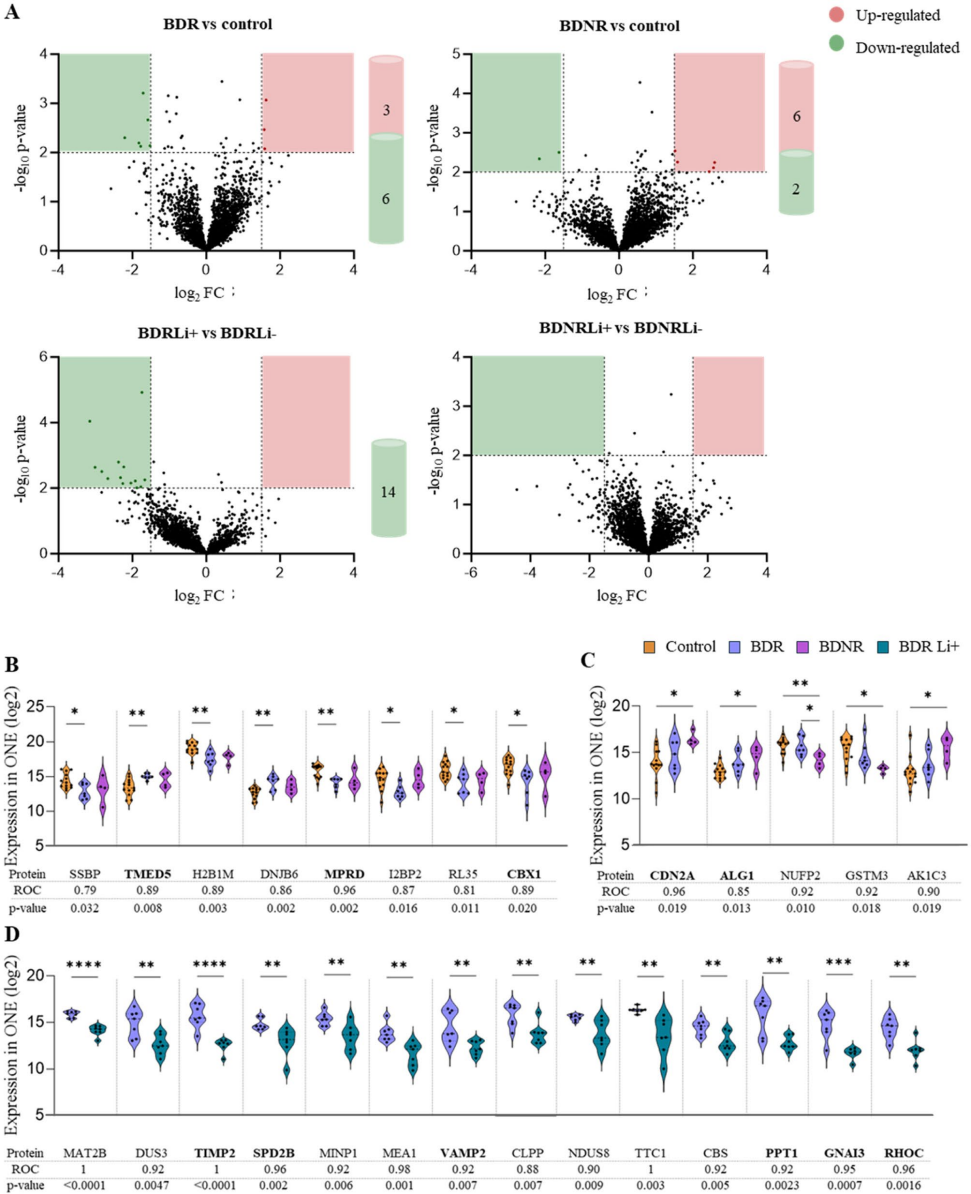
Differential Expressed Proteins (DEPs) and biomarker candidates.** A** Volcano plots showing differentially expressed proteins in four comparison groups. Each plot displays proteins that are significantly upregulated (red) or downregulated (green) based on a log₂ fold change (log₂FC) threshold of > 1.5 or < − 1.5 and a significance cutoff of -log_10_ p-value > 2. Black dots represent non-significant proteins.** B** DEPs uniquely identified in bipolar disorder responder (BDR) patient olfactory neuroepithelium cells compared to controls. **C** DEPs uniquely identified in bipolar disorder non-responder (BDNR) patient olfactory neuroepithelium cells compared to controls. **D** DEPs identified in BDR patient olfactory neuroepithelium cells following vitro lithium treatment. ONE: olfactory neuroepithelium cells; BDR: bipolar disorder patients responder to lithium; BDNR: bipolar disorder patients non-responder to lithium; Li+: treated with lithium in vitro; Li-: non-treated with lithium in vitro; ROC: Receiver Operating Characteristic curve. Bold proteins: DEPs associated with altered biofunctions and having ROC > 0.8. p-values: *p < 0.005; **p < 0.01; ***p < 0.001; ****p < 0.0001

Second, the LFQ proteomic analysis revealed several proteins with differential expression between groups (defined by a log_2_FC > 1.5 for upregulation, log_2_FC < −1.5 for downregulation, and p-value < 0.01), as illustrated in the volcano plots (Fig. 3A). In the comparison between BDR and control groups, 9 proteins were differentially expressed, with 3 showing upregulation and 6 downregulation. In the comparison of ONE cells derived from BDNR and control groups, 8 proteins were differentially expressed, including 6 upregulated and 2 downregulated. Lithium treatment in vitro resulted in the differential expression of 14 proteins in BDR patients, all of which were downregulated. Conversely, no differentially expressed proteins were identified in BDNR-derived ONE cells following lithium treatment (Fig. 3A).

#### Potential markers of diagnosis and/or lithium response

From the total of DEPs identified, we further focused on those that presented statistically significant differences exclusively in BDR vs. control or BDNR vs. control (Fig. 3B and C, respectively). 8 DEPs were differentially expressed in BDR compared to control (SSBP, TMED5, H2B1M, DNJB6, MPRD, I2BP2, RL35 and CBX1; Fig. 3B), while 5 DEPs were identified in BDNR cells (CDN2A, ALG1, NUFP2, GSTM3, AK1C3; Fig. 3B). AUROC analysis confirmed the diagnostic potential of all these DEPs, with values exceeding 0.80, except for the protein SSBP with AUROC = 0.79 (Fig. 3B-C).

Regarding the impact of in vitro lithium treatment (Fig. 3D), we identified 14 DEPs, all of them downregulated in BDR cells in response to lithium, compared to untreated BDR cells (MAT2B, DUS3, TIMP2, SPD2B, MNP1, MEA1, VAMP2, CLPP, NDUS8, TTC1, CBS, PPT1, GNAI3, RHOC; Fig. 3C). On the other hand, no significant changes were observed in BDNR lithium-treated cells compared to untreated BDNR. This striking lack of lithium-induced changes in protein expression in BDNR cells is a critical finding, potentially indicating a fundamental biological resistance to lithium’s actions that underlies their clinical non-responsiveness. Notably, all the proteins that exhibited lithium-induced changes specifically in BDR cells, had AUROC values greater than 0.85 (Fig. 3D), so they could be considered as potential markers of lithium response.

#### Biomarker analysis and correlations with cellular parameters

**Fig. 4:**
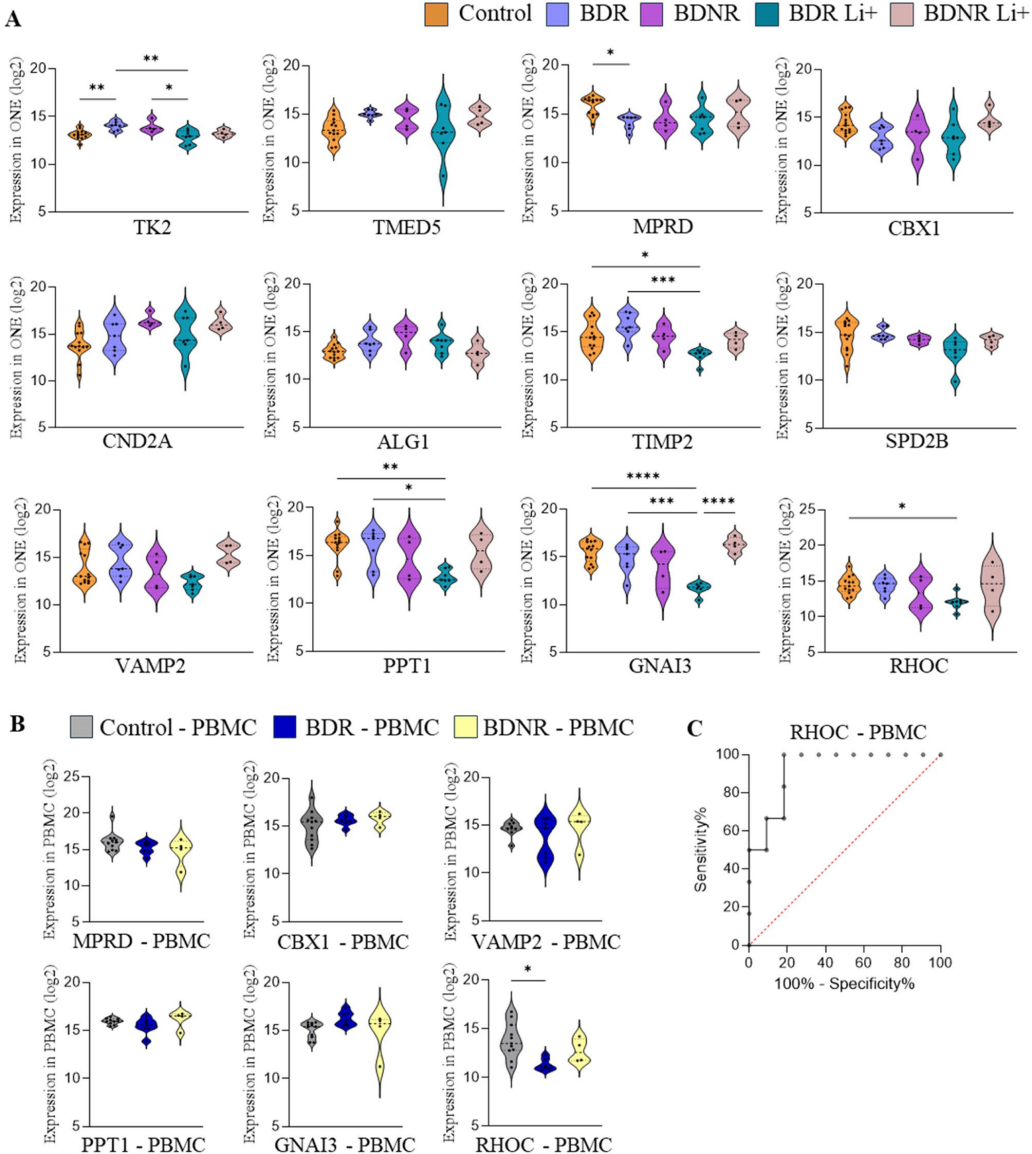
Potential biomarkers and correlations to cellular parameters. **A** Quantification and comparison between all experimental groups of selected proteins expressed in olfactory neuroepithelial cells. **B** Quantification and comparison between all experimental groups of selected proteins expressed in PBMC.** C** ROC analysis of RHOC protein expression level in PBMC, considering control and BDR groups. BDR: bipolar disorder patients responder to lithium; BDNR: bipolar disorder patients non-responder to lithium; Li+: treated with lithium in vitro; Li-: non-treated with lithium in vitro. PBMC: peripheral blood mononuclear cells; ROC: Receiver Operating Characteristic curve. *p < 0.05; **p < 0.01; ***p < 0.001

Based on the DEPs involved in disrupted cellular functions identified by IPA (Fig. 2) and the biomarker candidates described above (Fig. 3), we further analyzed their expression profiles across different experimental groups to identify potential biomarkers of lithium response (Fig. 4).

The mitochondrial protein Thymidine Kinase 2 (TK2) protein was found among the DEPs connected to the “cell morphology” changes reported (Fig. 2). This protein was overexpressed in BDR cells compared to control and uniquely downregulated in response to Lithium (BDRLi + vs. BDR), highlighting its potential as a biomarker of lithium treatment response. Other potential markers of lithium response were identified, that showed a specific decrease in expression in lithium-treated BDR cells (MPRD, TIMP2, PPT1, GNAI3 and RHOC; Fig. 4A).

To assess their potential as peripheral biomarkers, the proteins were also analyzed in PBMCs from the same donors. Among the remaining proteins (excluded TK2, CND2A, ALG1, TIMP2 and SPD2B which were not detected in PBMCs), only RHOC showed a significant decrease in BDR patients compared to controls (Fig. 4B), reflecting the lithium-induced downregulation observed in BDR ONE cells (Fig. 4A). Additionally, RHOC expression levels in PBMCs exhibited a high AUROC value, suggesting their potential as a biomarker for BDR patients (AUROC = 0.92; Fig. 4C).

**Fig. 5:**
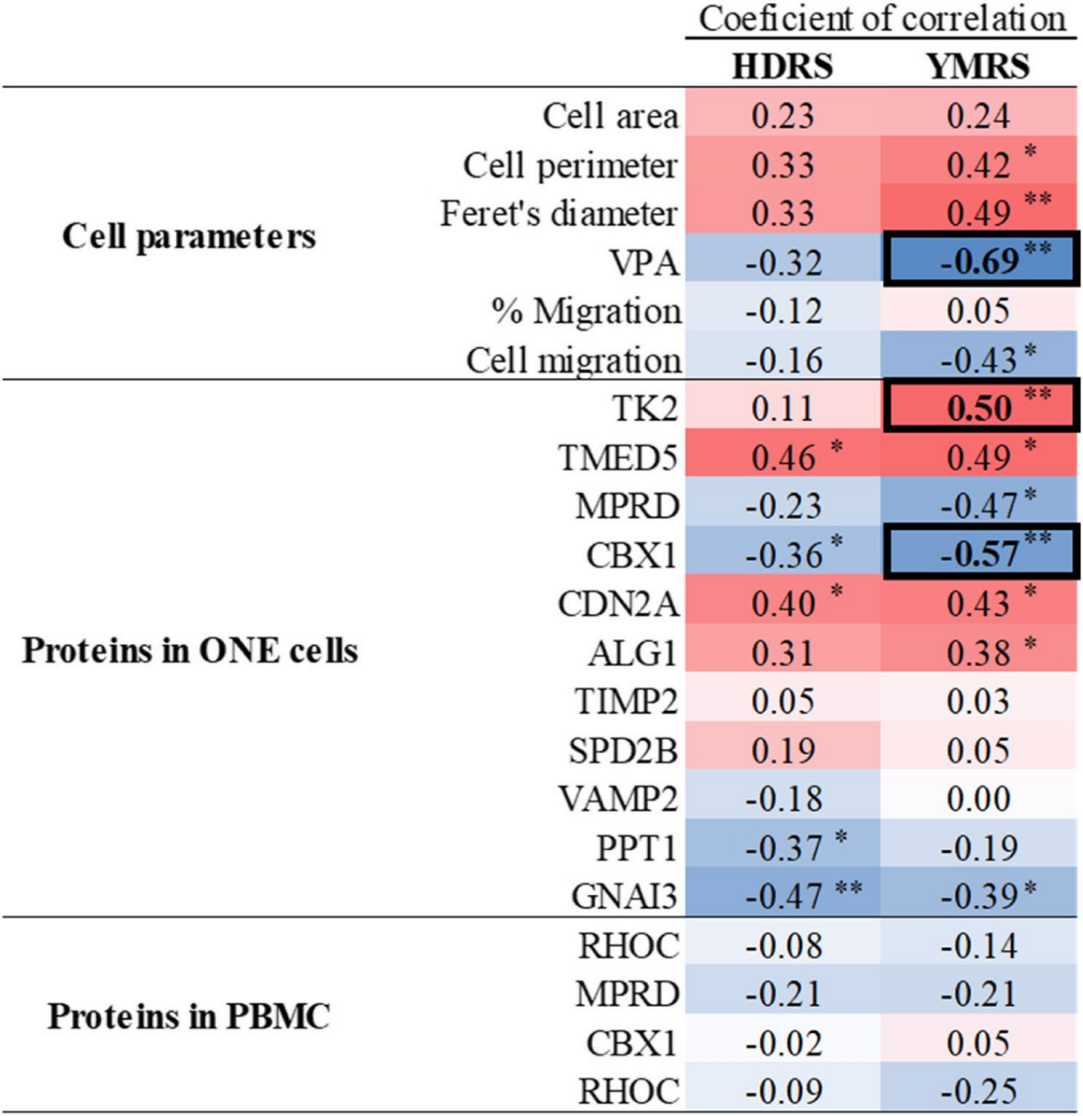
Correlation coefficients between cellular parameters and selected proteins with HRDS and YMRS scores. VPA: vinculin points of adhesion; HDRS: Hamilton Depression Rating Scale; YMRS: Young Mania Rating Scale. Correlation analysis: Pearson’s for data with normal distribution and Spearman’s for data without normal distribution.; *p < 0.05; **p < 0.01. Bold numbers: coefficient of correlation > 0.5

Subsequent analyses were conducted to explore potential correlations between altered factors in ONE cells—both at the level of cellular parameters and protein expression—and BD symptomatology, as assessed by the HRDS and YMRS scales (Fig. 5). Notably, significant correlations (correlation coefficient > 0.5) were identified for VPA levels, as well as the expression levels of TK2 and CBX1. Specifically, a decrease in VPA levels was associated with higher YMRS scores, indicating more severe manic symptomatology. Regarding protein expression, TK2 levels exhibited a positive correlation with YMRS scores, suggesting that elevated TK2 expression was linked to greater manic symptom severity. In contrast, CBX1 expression levels showed a negative correlation with YMRS scores, implying that lower CBX1 levels were associated with more pronounced manic symptoms. No other analyzed parameters demonstrated a correlation coefficient exceeding 0.5 (Fig. 5).

## Discussion

This study expands on the work of Delgado-Sequera et al. (2024b), which investigated differential changes in BD ONE cells relative to a control population. Here, we take this further by analyzing the effects of lithium treatment and the mechanistic differences between BDR- and BDNR-derived cells. Our goal is to identify distinct biological characteristics of each group and uncover potential targets linked to their clinical response to lithium.

In agreement with the neurodevelopmental hypothesis of BD, previous research has demonstrated cytoarchitectural abnormalities in post-mortem brains of BD patients (Gigase et al. 2019), with cytoskeletal dysfunction implicated through proteomic analyses of the human prefrontal cortex (English et al. 2009). These findings align with our data from ONE cells derived from BD patients and cultured in vitro. Specifically, labelling microtubules with β-III-tubulin revealed altered microtubule assembly, as evidenced by a greater unstained area relative to the total cell area in ONE cells from BD patients (Solís-Chagoyán et al. 2013). Furthermore, our previous study (Delgado-Sequera et al. 2024b) identified morphological alterations in BD ONE cells, characterized by a larger and more elongated shape. When stratifying the BD population based on lithium response, we found that these altered patterns were specifically observed in the BDNR group, which remained unchanged after lithium treatment in vitro. These data clearly align with biofunctional analysis from proteomics where robust changes were observed in “cell morphology” and “cellular assembly and organization” in BDNR compared to control cells where the protein CDN2A (cyclin-dependent kinase inhibitor 2 A) was highlighted in these functions, as well as in “cell-to-cell signaling and interaction” and “cellular movement”. Cyclin-dependent kinase inhibitors play multifaceted roles in the CNS, from cell cycle regulation to neuroprotection and modulation of neuronal processes. Interestingly, minor proteomic changes were produced by in vitro lithium treatment in this group aligning with the idea that lithium does not robustly impact the BDNR group. Given CDN2A’s specific differential expression in BDNR cells and its prominent role in altered cellular functions like morphology, its resistance to in vitro lithium modulation is particularly noteworthy, suggesting it may contribute to the inherent biological characteristics distinguishing BDNR patients. Therefore, from a therapeutic perspective, it would be worthwhile to explore the potential of cyclin-dependent kinase inhibitors or microtubule-stabilizing agents for BDNR patients. Supporting this approach, microtubule-targeting drugs are currently under development for treating neurodegenerative and psychiatric disorders, including schizophrenia and mood disorders (Varidaki et al. 2018).

We conducted a focal adhesion assay and found that ONE cells from both BDR and BDNR patients exhibited reduced adhesion capacity compared to control cells. Remarkably, this deficiency in focal adhesion points was reversed in both patient groups following in vitro lithium treatment. These findings highlight diminished focal adhesion as a hallmark feature of BD ONE cells. Furthermore, a lower number of focal adhesions in ONE cells was associated with higher YRMS scores, indicating greater severity of manic symptoms. Similar deficits have been described in schizophrenia-derived ONE cells (Fan et al. 2013), suggesting that impaired focal adhesion may be a shared characteristic of ONE cells in both BD and schizophrenia. Interestingly, recent integrative analyses combining GWAS and transcriptomic data from iPSC-derived neurons identified focal adhesion as one of the most significantly altered functions distinguishing BDR from BDNR (Niemsiri et al. 2024). Thus, our findings differ from patterns observed in iPSC-derived neurons, emphasizing potential model-specific differences. These differences may stem from divergent cell origins and differentiation states between ONE cells and iPSC-derived neurons, as shown in Niemsiri et al. (2024); Delgado-Sequera et al. (2024a). In addition to focal adhesion, we evaluated migration capacity. While no differences were observed between groups a fter 24 h, a significant reduction in the number of BD ONE cells migrating into the wound area was evident, particularly in the BDR group. This finding suggests that distinct proteins related to cell migration may underlie the observed effects in BDR cells compared to BDNR, as indicated by proteomic analyses of “Cellular Movement.” Although lithium treatment tended to enhance migration in BDR cells, this effect did not reach statistical significance. We also assessed cell proliferation and consistently with previous findings (Delgado-Sequera et al. 2024b), ONE cells from both BDR and BDNR patients showed no alterations in proliferation, in contrast to the changes reported for iPSC-derived neurons (Delgado-Sequera et al. 2024a). Therefore, our results reveal significant differences between BDR and BDNR ONE cells. BDNR cells exhibited pronounced alterations in cell morphology, cytoskeletal organization, and adhesion capacity, whereas BDR cells primarily showed deficits in adhesion capacity and migration. Notably, in vitro lithium treatment restored adhesion capacity in both groups.

Proteomics evaluation in BDR group, showed biofunctions predicted to be altered like “cell morphology” and “cellular assembly and organization”, as result of proteins differentially expressed such as the proteins TK2 (Thymidine Kinase 2), CBX1(Chromobox 1), MPRD (membrane progesterone receptor delta) and TMED5 (Transmembrane emp24 domain-containing protein 5) respectively. Regarding the protein CBX1, given its role in epigenetic regulation (Fang et al. 2022), it is possible that this protein could indirectly influence psychiatric disorders like BD through its effects on gene expression. Indeed, it is particularly noteworthy that we found a correlation between decreased CBX1 expression levels in ONE cells and higher scores on the manic YRMS scale. Similarly, MPRD appears to play a role in BD, particularly in relation to neurosteroid signalling (Carta et al. 2012) and mood regulation. This is significant because in women with BD during euthymia, plasma concentration of allopregnanolone (which binds to MPRD) is elevated in the premenstrual period compared to controls. This suggests a potential role for MPRD in mood stabilization (Carta et al. 2012). Regarding TMED5, it functions in conjunction with other TMED family proteins, forming complexes that are crucial for maintaining the stability and proper functioning of the early secretory pathway and Golgi apparatus which are essential for protein processing and trafficking in neurons (Zhou et al. 2023). Thus, these findings underscore significant differences in molecular and cellular pathways between BDNR and BDR groups. For BDNR patients, the lack of response to lithium treatment may be attributed to the unique upregulation of CDN2A. In BDR patients, DEPs proteins such as CBX1, MPRD, and TMED5, were associated with pathways related to epigenetics, neurosteroid signalling, and vesicle trafficking, offering insights into lithium’s therapeutic effects.

We next investigated the effects of in vitro lithium treatment on BDR and BDNR cells, revealing distinct proteomic alterations. In BDR ONE cells, lithium significantly reduced the expression of several DEPs, including GNAI3 (G Protein Subunit Alpha I3), TIMP2 (Tissue Inhibitor of Metalloproteinases 2), and RHOC (Rho-related GTP-binding protein RhoC). These proteins are associated with key biological functions such as **“**cell morphology”, “cellular assembly and organization”, “cell-to-cell signaling and interaction”, and “cellular movement”. Notably, lithium treatment had minimal or no effect on BDNR cells, particularly in functions like “cell morphology”, underscoring its selective impact on BDR ONE cells and highlighting its nuanced role in cellular processes. Among the proteins affected, GNAI3 was significantly decreased in BDR cells following lithium treatment. As an inhibitory alpha subunit of a G protein complex, GNAI3 mediates signal transmission from G protein-coupled receptors (GPCRs) to various cellular effectors. Alterations in G protein-related genes and signalling have been implicated in BD. For instance, GNAI1, another inhibitory alpha subunit, has been found to be upregulated in the anterior cingulate cortex of BD patients (Tomita et al. 2013). Another key protein significantly diminished by lithium in BDR ONE cells is TIMP2, a multifunctional regulator of extracellular matrix remodelling and various cellular processes. Interestingly, cerebrospinal fluid levels of TIMP-2 are significantly elevated in BD patients compared to controls, even after adjusting for age and sex (Jakobsson et al. 2015). However, serum levels of TIMP-2 show no significant differences between these groups (Jakobsson et al. 2015), and it was undetectable in PBMC samples in our study. This suggests that TIMP-2 may play a role primarily in central nervous system processes in BD. Additionally, TIMP-2 interacts with matrix metalloproteinases (MMPs), and lithium treatment has been shown to influence MMP activity, modulating cellular senescence (Struewing et al. 2009) and the MAPK/Erk-1/2 pathway (Haupt et al. 2020). Lithium treatment also reduced RHOC expression in BDR cells. RHOC is a member of the Rac subfamily of Rho GTPases, which play a crucial role in cytoskeletal regulation (Ridley 2006). Dysregulation of the Rho-ROCK pathway, which involves Rho GTPases, has been linked to neuronal cell loss, impaired synaptic function, and cytoskeletal abnormalities in central nervous system disorders (Wong et al. 2023). Lithium may exert its effects through Rho-mediated mechanisms, as it is a GSK3β inhibitor, and GSK-3 activation—known to regulate neurite retraction—is mediated by Rho signaling (Laura Sayas et al. 2002). Proteomic data from PBMC samples further support this finding, as RHOC expression was significantly downregulated in the BDR group, reinforcing its potential role in BD pathophysiology and lithium response. These effects are likely linked to lithium’s known modulation of intracellular signaling pathways, including its inhibition of GSK3β and interference with Rho-mediated cytoskeletal dynamics, which may underlie the observed changes in morphology and adhesion.

Proteomic functional analysis also identified PPT1 (Palmitoyl-Protein Thioesterase 1) and VAMP2 (Vesicle-Associated Membrane Protein 2) as key regulators of critical cellular processes, including “cell morphology”, “assembly and organization”, “cell-to-cell signalling and interaction”. Notably, these proteins were significantly reduced by lithium treatment in ONE cells derived from individuals with BDR. PPT1, which plays a crucial role in neuronal morphology and function (Sapir et al. 2019), has been linked to schizophrenia, with higher enzymatic activity correlating with disease severity (Wu et al. 2019). Regarding VAMP2, although a study on German BD patients found no significant association between VAMP2 genetic variants and BD (Jamra et al. 2008), mutations in VAMP2 have been implicated in various neurodevelopmental disorders, including intellectual disability, autism spectrum disorder, and epilepsy (Simmons et al. 2020). Additionally, lithium treatment has been shown to significantly reduce aberrant VAMP2-positive puncta along neuronal processes in cells transfected with the DISC1 breakpoint mutant (Lee et al. 2011)—a gene strongly implicated in BD (Carter 2007; Macgregor et al. 2004; Maeda et al. 2006; Perlis et al. 2008). As previously noted, the mitochondrial enzyme TK2 is overexpressed in BDR cells, uniquely downregulated by lithium treatment, and correlated with manic symptom severity. This finding aligns with prior evidence suggesting mitochondrial dysfunction in BD (Mertens et al. 2015). While not universally accepted, some studies suggest that the normalization of specific mitochondrial alterations underlies lithium’s favorable therapeutic response (Stacey et al. 2018). Overall, these results emphasize lithium’s selective impact on BDR cells, significantly modulating proteins involved in synaptic function, neurotransmitter release, and mitochondrial activit**y**, which are disrupted in BD. Conversely, the lack of response in BDNR cells supports the hypothesis of distinct molecular mechanisms underlying lithium responsiveness. Nevertheless, other factors may contribute to BDNR resistance, including prior lithium exposure, epigenetic modifications, or long-term cellular adaptations not captured in our in vitro model. Future research should further explore these protein targets to refine diagnostic markers and therapeutic strategies for bipolar disorder.

The results of this preliminar study should be considered in light of its strengths and limitations. First of all, the small sample size for each experimental group may affect the generalizability and the statistical power of our findings. This limited power likely contributes to the relatively low number of statistically significant differentially expressed proteins identified, and as such, these results must be interpreted with caution. Besides, the lack of longitudinal follow-up limits our ability to assess dynamic responses over time. Future studies should address these aspects through larger cohorts and repeated sampling. Second, while our study was exploratory and aimed to identify a broader range of relevant proteins, we acknowledge that the absence of a formal multiple testing correction, such as FDR, increases the risk of false positives, despite our use of a stringent uncorrected p-value threshold of 0.01 and an additional 1.5-fold change filter. Additionally, there is a scarcity of comparable studies, as most previous research has focused on lymphocytes, iPSC-derived neurons (Niemsiri et al. 2024), or post-mortem brain tissue. Although earlier research has explored potential biomarkers of neuropsychiatric disorders using ONE cells (Barrera-Conde et al. 2021; Benítez-King et al. 2016; Fan et al. 2013), stratification based on treatment response was unexamined. Despite these limitations, our findings reveal significant cellular and molecular differences between BDNR and BDR cells. To our knowledge, this is the first comprehensive proteomic analysis to characterize differentially expressed proteins between BDNR and BDR cells in response to lithium. The proteomic data underscore distinct molecular signatures associated with lithium responsiveness. Further research is needed to further investigate these pathways, enhance our understanding of lithium responsiveness, and advance diagnostic and therapeutic strategies for BD. These findings provide important insights for personalized medicine in bipolar disorder by identifying molecular differences between lithium responders and non-responders. Future studies with larger cohorts and in vivo validation are needed to develop targeted therapies and improve clinical management. Additionally, these studies should control for potential confounding factors such as medication use, age, sex, and BMI to ensure more robust and generalizable findings.

## Data Availability

Additional datasets from this study are available from the corresponding author upon reasonable request.
